# The Fate of Pre-Existing L5-S1 Degeneration following Oblique Lumbar Interbody Fusion of L4-L5 and Above

**DOI:** 10.3390/jcm12237463

**Published:** 2023-12-01

**Authors:** Dong-Ho Kang, Ji Hwan Kim, Bong-Soon Chang, Hyoungmin Kim, Dongook Kim, Sanghyun Park, Seong Hwa Hong, Sam Yeol Chang

**Affiliations:** Department of Orthopedic Surgery, Seoul National University Hospital, Seoul National University College of Medicine, 101 Daehangno, Jongno-gu, Seoul 03080, Republic of Korea; kang9451@snu.ac.kr (D.-H.K.); 7a794@snuh.org (S.P.); hsh54321@snu.ac.kr (S.H.H.)

**Keywords:** oblique lumbar interbody fusion (OLIF), adjacent segment disease (ASD), facet effusion, severe disc degeneration, severe fatty degeneration

## Abstract

Background: Previous studies have identified various risk factors for adjacent segment disease (ASD) at the L5-S1 level after fusion surgery, including preoperative sagittal imbalance, longer fusion, and preoperative disc degeneration. However, only a few studies have explored the risk factors for ASD at the L5-S1 level after oblique lumbar interbody fusion (OLIF) at the L4-L5 level and above. This study aimed to identify the risk factors for symptomatic ASD at the L5-S1 level in patients with pre-existing degeneration after OLIF at L4-L5 and above. Methods: We retrospectively reviewed the data of patients who underwent OLIF at L4-L5 and above, with a minimum follow-up period of 2 years. Patients with central stenosis or Lee grade 2 or 3 foraminal stenosis at L5-S1 preoperatively were excluded. Patients were divided into ASD and non-ASD groups based on the occurrence of new-onset L5 or S1 radicular pain requiring epidural steroid injection (ESI). The clinical and radiological factors were analyzed. Logistic regression was used to identify the risk factors for ASD of L5-S1. Results: A total of 191 patients with a mean age ± standard deviation of 68.6 ± 8.3 years were included. Thirty-four (21.7%) patients underwent ESI at the L5 root after OLIF. In the logistic regression analyses, severe disc degeneration (OR (95% confidence interval (CI)): 2.65 (1.16–6.09)), the presence of facet effusion (OR (95% CI): 2.55 (1.05–6.23)), and severe paraspinal muscle fatty degeneration (OR (95% CI): 4.47 (1.53–13.05)) were significant risk factors for ASD in L5-S1. Conclusions: In this study, the presence of facet effusion, severe disc degeneration, and severe paraspinal muscle fatty degeneration at the L5-S1 level were associated with the development of ASD at L5-S1 following OLIF at L4-L5 and above. For patients with these conditions, surgeons could consider including L5-S1 in the fusion when considering OLIF at the L4-L5 level and above.

## 1. Introduction

Lumbar interbody fusion effectively treats various lumbar degenerative conditions but modifies the biomechanics of adjacent spinal segments, leading to increased motion and potential degenerative changes [1,2,3]. The increased motion and stress on the non-operated adjacent motion segments can cause adjacent segment degeneration, with symptomatic adjacent segment disease (ASD) reported in approximately 30% of post-fusion surgery patients within a decade [4]. The L5-S1 segment, a distinctive transitional junction with unique motion and biomechanical properties, can experience increased stress-induced degeneration due to its structural alignment and weight-bearing role [5,6]. The reported incidence of adjacent segment degeneration in L5-S1 level after lumbar fusion ranges from 4% to 28% [7,8,9,10,11]

Minimally invasive oblique lateral interbody fusion (OLIF) has the potential to reduce adjacent disc degeneration of L5-S1 after L4-L5 fusion by minimizing paraspinal muscle violation and restoring segmental lordosis more effectively than posterior (PLIF) and transforaminal (TLIF) lumbar interbody fusion [10,12]. However, technical challenges arise from dissecting obstructing vascular structures during OLIF at L5-S1 [13]. These technical challenges in cases without definitive indications for surgery at the L5-S1 level, such as mild foraminal stenosis without central stenosis, may influence the surgeon’s decision-making. In patients lacking clear surgical indications at the L5-S1 level, some exhibited disc, facet, and paraspinal muscle degeneration at the time of surgery, which could contribute to symptomatic ASD if L5-S1 is not fused, presenting surgeons with challenges in balancing surgical invasiveness and the risk of subsequent treatment.

Previous studies have identified various risk factors for ASD at the L5-S1 level after fusion surgery, including preoperative sagittal imbalance, longer fusion, and preoperative disc degeneration [14,15,16]. However, only a few studies have explored the risk factors for ASD at the L5-S1 level after OLIF at the L4-L5 level and above [10,17]. To reduce symptomatic ASD and related revision surgeries at L5-S1, it is crucial to identify patients at high risk. Therefore, in this study, among all pre-existing degenerative changes at the L5-S1 level, we aimed to identify the risk factors associated with developing symptomatic ASD at L5-S1 following OLIF at the L4-L5 level and above.

## 2. Materials and Methods

### 2.1. Patients

We conducted a retrospective review of consecutive patients who underwent circumferential minimally invasive oblique lateral interbody fusion (cMIS-OLIF) at the L4-L5 level and above using percutaneous pedicle screw instrumentation between August 2012 and December 2022. The institutional review board of Seoul National University Hospital (IRB reference number: 2304-016-1419) approved the current study, and the requirement for informed consent was waived because of its retrospective nature. The inclusion criteria were (1) a minimum of 2 years of follow-up, and patients with pre-existing (2) disc degeneration at the L5-S1 level, classified as Pfirrmann grade 1 or above [18], (3) facet degeneration at the L5-S1 level, classified as osteoarthritis grade 1 or above [19], and (4) pre-existing fatty degeneration of the paraspinal muscle at the L5-S1 level, classified as Goutallier grade 1 or above [20]. The exclusion criteria were (1) any preoperative central stenosis at the L5-S1 level, (2) preoperative foraminal stenosis higher than Lee grade 2 at the L5-S1 level [21], (3) preoperative lateral recess stenosis higher than Bartynski grade 2 at the L5-S1 level [22], (4) previous fusion surgery at the L5-S1 level, and (5) patients with congenital stenosis, malignancy, inflammatory disease, or infection.

### 2.2. Surgery

Most patients underwent cMIS-OLIF via an anterior retroperitoneal approach in the lateral decubitus position. A polyether-ether-ketone (PEEK) intervertebral cage filled with demineralized bone matrix was inserted into the intervertebral disc space after discectomy, increasing the posterior disc space height. Cages with 6° or 12° lordotic angles were implanted in all patients, positioned between the middle and anterior third of the disc space. We aimed to maximize disc height and lumbar lordosis (LL) without employing anterior column release (ACR) or posterior osteotomy. When ACR was omitted, cages with angles > 12° were avoided, as the limited disk space expansion caused a reduced contact area with the endplate, even when inserting a cage with an angle larger than the endplate. Patients were then repositioned to a prone position for posterior percutaneous pedicle screw insertion under intraoperative fluoroscopy. In the prone position, we employed a chest bar and two posts under both the anterior superior iliac spine to achieve optimal lumbar sagging to maximize LL. Screw insertion was performed in situ without any compression or distraction maneuvers.

### 2.3. Demographic Data and Primary Outcome

Demographic data, including patient age, sex, and body mass index (BMI), as well as preoperative diagnosis and treatment-related factors, such as the number of surgical levels, prior surgery history at the index surgical level, and perioperative complications, were obtained from electronic medical records and picture archiving and communication systems. The primary outcome measure was the occurrence of symptomatic ASD during the follow-up period after OLIF at the L4-L5 level and above. The ASD group included patients who experienced new-onset L5 or S1 radicular pain uncontrolled by pain medications for more than 3 months and required pain intervention, such as epidural steroid injection (ESI). In addition, the fusion extension at L5-S1 due to symptomatic ASD was also investigated. Fusion extension was contemplated for patients exhibiting radiological deterioration in the adjacent segment that was unresponsive to pharmacotherapy and tri-monthly ESIs.

### 2.4. Radiological Assessment

As for the radiological assessment, preoperative lumbosacral standing X-ray images and axial and sagittal T1- and T2-weighted magnetic resonance imaging (MRI) images were reviewed by two authors who were unaware of the clinical outcomes. In cases of disagreement regarding categorical variables, arbitration by a third author to resolve the discrepancy was conducted. In preoperative plain radiographs, deep-seated L5, calcified L5-S1 disc, decreased disc height at the L5-S1 level, instability in L5-S1, degenerative spondylolisthesis in L5-S1, flexion–extension range of motion (ROM) of the L5-S1 disc space, and preoperative and postoperative sagittal parameters such as pelvic incidence (PI), LL, sacral slope (SS), pelvic tilt (PT), and sagittal vertical axis (SVA) were investigated. The preoperative and postoperative lordosis distribution index (LDI) were calculated using the following formula: $\frac{L4-S1\;\mathrm{lordosis}}{L1-S1\;\mathrm{lordosis}}$ × 100 [23]. Given that patients with a lumbopelvic mismatch exceeding 15° face a significantly increased risk of ASD-related reoperation [24], we defined a PI-LL mismatch as a value of ≥15° on standing whole-spine lateral radiographs. Instability was characterized as a ROM exceeding 10° or vertebral body translation exceeding 3 mm in the L5-S1 disc space when comparing flexion and extension lumbar lateral X-rays [25]. Deep-seated L5 was defined by the L5 pedicles as greater than 10 mm below the intercrestal line [26]. Disc vacuum was evaluated using preoperative lateral plain radiographs, including flexion, neutral, and extension views. In preoperative MRI images, we investigated the preoperative Pfirrmann grade of the L5-S1 disc [18], osteoarthritis grading of L5-S1 facet joints [19], the Goutallier grade of the paraspinal muscle at the L5-S1 disc level [20], the presence of facet cysts, the presence of facet effusion in L5-S1 facet joints [27], the presence of preoperative Bartynski grade 1 lateral recess stenosis at the L5-S1 level [22], and the presence of preoperative Lee grade 1 foraminal stenosis at the L5-S1 level. According to Chaput et al.’s classification, facet joint effusion is identified as a measurable (≥1 mm) curvilinear high signal, akin to cerebrospinal fluid signals, on axial T2-weighted MRI scans [27]. The bilaterality of facet effusion was also collected. In patients with supplementary MRI examinations during the follow-up period, the progression of central and foraminal stenosis at the L5-S1 level was assessed using Schizas and Lee grades [21,28]. The progression of lateral recess stenosis at the L5-S1 level was assessed using Bartynski grades [22].

### 2.5. Statistical Analysis

Differences in continuous data between the ASD and non-ASD groups were assessed using Student’s *t*-test or the Kruskal–Wallis test, whereas differences in categorical data were evaluated using the chi-square test or Fisher’s exact test for statistical analysis. Logistic regression analysis was performed to identify the factors associated with symptomatic ASD at L5-S1. To optimize variable selection and reduce the risk of omitting potentially significant factors, variables that demonstrated a significant association (*p* < 0.20) in the univariate logistic regression analysis were included in the multivariate logistic regression model. The backward elimination method was used to compute the odds ratios (OR) and 95% confidence intervals (CI) of the variables to predict symptomatic ASD. The incidence and timing of symptomatic ASD were analyzed using the Kaplan–Meier method. The estimated median survival time, denoting the juncture at which half the study cohort is projected to have survived, was calculated from the Kaplan–Meier survival curve. Interaction effects between risk factors were assessed via mediation analysis using Process Macro in SPSS. Statistical analyses were performed using IBM SPSS Statistics (version 25.0; IBM, Armonk, NY, USA).

## 3. Results

### 3.1. Demographic Data and Primary Outcome

**Figure 1:**
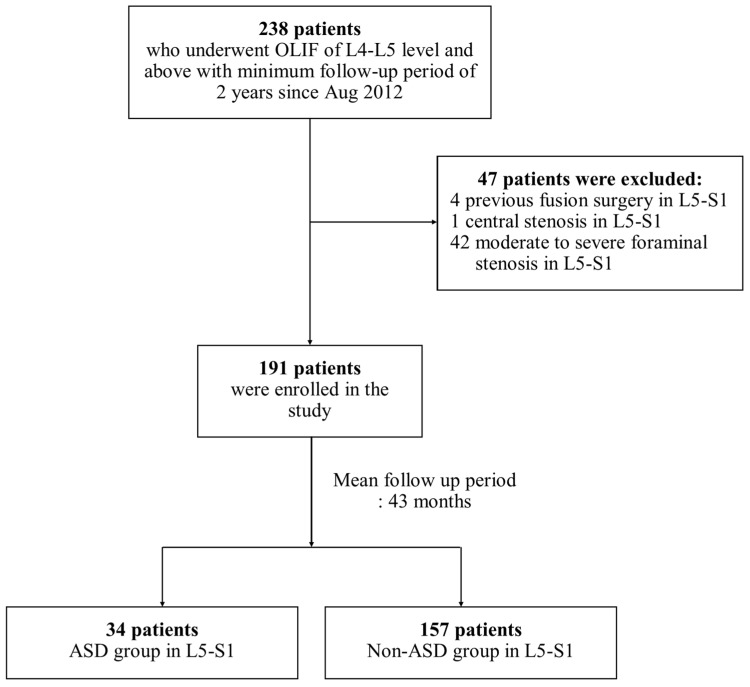
Flowchart of participant recruitment. OLIF, oblique lumbar interbody fusion; ASD, adjacent segment disease.

A total of 191 patients with a mean age ± standard deviation (SD) of 68.6 ± 8.3 years were enrolled in this study (Figure 1). The mean follow-up duration was 42.8 ± 17.5 months. Three patients with lumbarized S1 vertebrae were included, and none had sacralized L5 vertebrae. Thirty-four (21.7%) patients received ESI for L5 or S1 radicular pain after 19.9 ± 20.9 months following OLIF at the L4-L5 level and above. Among patients who received ESI, six (17.6%) never returned to our clinic after the first ESI treatment. The mean follow-up period after ESI was 25.2 ± 20.6 months. Three (1.6%) patients underwent fusion extension surgery for symptomatic ASD at the L5-S1 level after 34.2 ± 14.2 months following OLIF of the L4-L5 level and above. Table 1 summarizes the demographic characteristics of the patients in the ASD and non-ASD groups, which showed no significant differences in age, sex, BMI, follow-up period, preoperative diagnosis, and the number of levels at which OLIF was performed between the two groups.

### 3.2. Radiological Parameters between ASD Group and Non-ASD Group

All radiological parameters from the preoperative plain radiographs showed no significant difference between the two groups, including sagittal profiles such as PI, LL, SS, PT, LDI, PI minus LL, and SVA (Table 2). Since we excluded any preoperative central stenosis at the L5-S1 level and preoperative foraminal stenosis higher than Lee grade 2 at the L5-S1 level, only one patient with degenerative spondylolisthesis at the L5-S1 level was included in our study, and none had spondylolytic spondylolisthesis at the L5-S1 level. The preoperative flexion–extension ROM in the L5-S1 disc of the ASD group was slightly larger than that in the non-ASD group; however, the difference was not significant (*p* = 0.114). On the MRI, the presence of Lee grade 1 foraminal stenosis, and the presence of Bartynski grade 1 lateral recess stenosis, Pfirrmann grade in the L5-S1 disc, and the preoperative grade of facet arthrosis in the L5-S1 facet joint showed no significant differences between the two groups (Table 2). However, the proportion of patients with facet effusion ni the L5-S1 facet joints was significantly higher in the ASD group (*p* = 0.013). The ASD group also showed more severe paraspinal muscle fatty degeneration with a higher Goutallier grade at the L5-S1 disc level than the non-ASD group (*p* = 0.014).

### 3.3. Progression of Spinal Stenosis in Follow-Up MRI

In cases of worsening back pain or new-onset radicular symptoms during the follow-up period, a follow-up MRI was contemplated after patient consultation. Among all patients, 112 (58.6%) had follow-up MRI imaging at 24.5 ± 18.1 months after OLIF. In the follow-up MRI, five (4.5%) patients developed central stenosis at the L5-S1 level, and 46 (41.1%) cases showed progression or the development of foraminal stenosis at the same level, assessed using the Schizas and Lee grading systems, respectively. Additionally, 10 (5.2%) patients experienced an increase of at least one grade in lateral recess stenosis at L5-S1, as per the Bartynski scale. The prevalence of new or progressed foraminal stenosis was significantly higher in the ASD group than in the non-ASD group (56.7% vs. 35.4%, *p* = 0.042). While the ASD group also showed a higher occurrence of new central stenosis, this increase was not statistically significant (10.0% vs. 2.4%, *p* = 0.086). Similarly, the rate of new or progressed lateral recess stenosis was greater in the ASD group, though the difference was not statistically significant (16.7% vs. 6.1%, *p* = 0.128).

### 3.4. Risk Factors for Symptomatic ASD

In the multivariate logistic regression analyses, severe disc degeneration of Pfirrmann grades IV and V in L5-S1 disc (OR (95% CI): 2.65 (1.16–6.09)), the presence of facet effusion at L5-S1 level (OR (95% CI): 2.55 (1.05–6.23)), and severe paraspinal muscle fatty degeneration of Goutallier grades 3 and 4 (OR (95% CI): 4.47 (1.53–13.05)) in paraspinal muscle at the L5-S1 level were significant risk factors for symptomatic ASD (Table 3, full-version in Table S1).

### 3.5. Interaction Effects between Identified Risk Factors

In our study, patients with facet effusion were more associated with severe disc degeneration of Pfirrmann grades IV or V than those without facet effusion (39.5% vs. 24.3%, respectively, *p* = 0.050). No significant difference in the prevalence of severe fatty degeneration was noted between patients with and without facet effusion (7.0% and 13.5%, respectively, *p* = 0.246) or severe disc degeneration (11.3% and 12.3%, respectively, *p* = 0.849). Mediation analysis confirmed the three risk factors as independent contributors to symptomatic ASD (Table S2).

### 3.6. Survival Analysis of Identified Risk Factors

**Figure 2:**
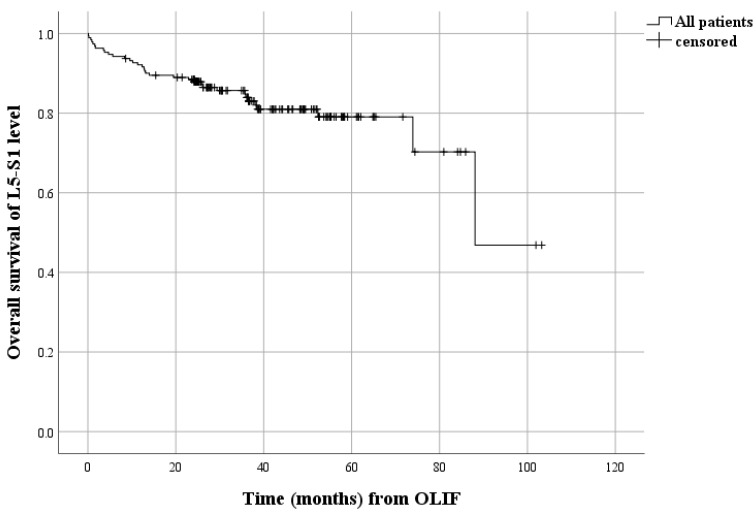
Kaplan–Meier survival curve of all patients showing occurrence of symptomatic ASD. ASD, adjacent segment disease.

**Figure 3:**
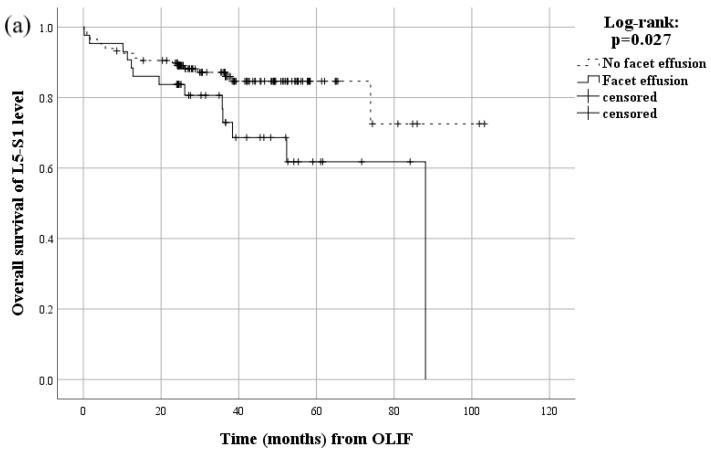

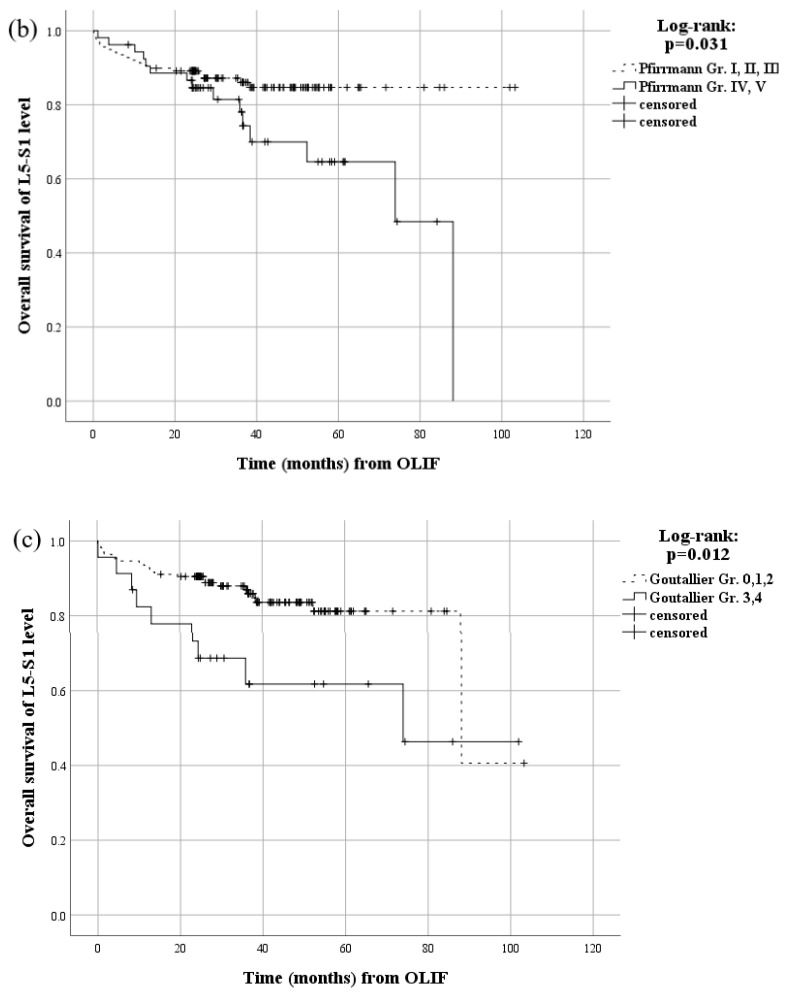
Kaplan–Meier survival curve showing occurrence of symptomatic ASD according to (**a**) presence of facet effusion in L5-S1 facet joint, (**b**) severe disc degeneration, and (**c**) severe paraspinal muscle fatty degeneration. ASD, adjacent segment disease.

In the survival analysis, patients with symptomatic ASD showed an estimated median survival of 88.1 months (Figure 2). The cumulative 24- and 60-month overall survival rates among all patients were 88.4% and 78.9%, respectively. A significant difference was observed in the overall survival of the L5-S1 level between the group of patients with pre-existing facet effusion and those without (*p* = 0.027) (Figure 3a). This difference was also evident when comparing patients with and without severe disc degeneration of Pfirrmann grades IV or V at the L5-S1 level (*p* = 0.031, Figure 3b), as well as between patients with and without severe fatty degeneration of Goutallier grades 3 or 4 in the paraspinal muscle at the L5-S1 level (*p* = 0.012, Figure 3c).

## 4. Discussion

In this study, the presence of facet effusion in the L5-S1 facet joint, severe disc degeneration of Pfirrmann grades IV and V in the L5-S1 disc, and severe fatty muscle degeneration at the L5-S1 disc level were significantly associated with symptomatic ASD occurrence following OLIF at the L4-L5 level and above.

Thirty-four (21.7%) patients received ESI for L5 or S1 radicular pain, and only three (1.6%) underwent fusion extension surgery for symptomatic ASD at the L5-S1 level following OLIF at the L4-L5 level and above. The observed low surgical rate for symptomatic ASD may be attributable to the loss to follow-up of patients who received initial ESI. Specifically, 17.6% of these patients did not return to our clinic after ESI, likely finding secondary opinions from other hospitals due to unsatisfactory symptom relief through medication and injections and potentially seeking care elsewhere. Consequently, the surgical rate may be underestimated.

A previous study reported that segmental instability is associated with the development of ASD following lumbar fusion surgeries [29]. However, our findings suggest that dynamic instability, as assessed via radiographic measurement, did not correlate with symptomatic ASD at the L5-S1 level following OLIF at the L4-L5 level and above. This could be attributed to the distinct segmental motion and biomechanical characteristics of the L5-S1 segment, which separates it from the other lumbar segments [5,6]. Sabnis et al. demonstrated that the L5-S1 segment undergoes re-stabilization in cases of advanced degeneration of the intervertebral disc and facet joints [30]. Conversely, this phenomenon of re-stabilization was not observed in the upper spinal segments. Contrary to upper spinal segments, the L5-S1 level often gains stability with progressive degenerative changes in adjacent structures, suggesting that traditional criteria for dynamic instability may not reliably predict ASD at this level. In our study, the presence of facet effusion, often considered an indicator of segmental instability, was a significant risk factor for symptomatic ASD at the L5-S1 level. Our result is consistent with Chaput et al.’s study, demonstrating that facet joint effusion is an independent risk factor for degenerative spondylolisthesis. They showed that if supine MRI shows no detectable spondylolisthesis and effusion is absent, the likelihood of observing abnormal sagittal plane translation on flexion–extension films is low [27]. Consequently, the prevailing instability criteria may not be applicable at the L5-S1 level, suggesting that facet effusion could serve as a better index of instability at the L5-S1 level. Facet opening, assessed via CT, is also indicative of segmental instability [31]. However, routine preoperative CT was not performed in our hospital, limiting the evaluation of facet opening. According to Yamada, the concordance between facet joint opening and effusion is limited [32]. Hence, in future studies at institutions where preoperative CT is standard, evaluating facet joint opening is imperative.

Pre-operative disc degeneration has been identified as a significant risk factor for symptomatic ASD after lumbar interbody fusion in previous studies [24,33]. Our logistic regression analysis also showed that preoperative disc degeneration of Pfirrmann grades IV and V was significantly associated with symptomatic ASD after OLIF at the L4-L5 level and above. However, Conaway et al. reported that preoperative disc height ratio and Pfirrmann grading of the L5-S1 level was not predictive of worse clinical outcomes after isolated L4-L5 fusion [34]. This discrepancy could be explained by the distinct degenerative and kinematic properties of the L5-S1 segment compared to the upper lumbar motion segments, which result in re-stabilization in the advanced degeneration of intervertebral disc and facet joints [30,34]. Our results also showed that anterior and posterior disc height were not significant risk factors for symptomatic ASD at the L5-S1 level (Table S1).

Severe preoperative paraspinal muscle fatty degeneration of Goutallier grades 3 and 4 is also a significant risk factor for symptomatic ASD development. These findings are consistent with those of a previous study showing that the degree of fatty infiltration of the multifidus muscle at L4-L5 and L5-S1 is an important risk factor for symptomatic ASD [35]. These results indicate that the paraspinal muscles at the lower lumbar level play a significant role in the development of symptomatic ASD and emphasize the importance of preserving the paraspinal muscles during lumbar fusion surgeries. Backhauß and colleagues reported a significant association between fatty degeneration of the autochthonous spinal musculature and non-traumatic fractures of the lower thoracic spine [36]. Dohzono et al. demonstrated that a reduced paraspinal muscle area correlates with shorter overall survival in patients with spinal metastases [37]. These findings imply that the condition of the paraspinal musculature is crucial for various spinal pathologies.

Given the potential overlap or interaction effects among the three risk factors for symptomatic ASD, mediation analysis was conducted. In our study, the higher prevalence of severe disc degeneration among patients with facet effusion (39.5% vs. 24.3%, *p* = 0.050) suggests a potential interaction effect between these variables. However, mediation analysis revealed that facet effusion and severe disc degeneration were independent contributors to symptomatic ASD. These findings indicate that each of the three identified variables is an independent risk factor for symptomatic ASD.

Preoperative and postoperative sagittal parameters, such as PI-LL mismatch, have been reported as independent risk factors for ASD [29,38,39,40,41]. However, in this study, these parameters were not significant risk factors according to the logistic regression analysis. A plausible explanation for this discrepancy may be that OLIF, by virtue of employing a larger-sized cage, potentially has superior advantages for LL restoration compared with PLIF or TLIF [12,13,42,43,44,45]. The observed discrepancy could result from differences in the composition of Roussouly types between earlier studies and our study cohort. The data from Table 2 indicate an average PI of approximately 54, with a standard deviation around 9, suggesting that the majority of included patients align with higher Roussouly types [46]. This is consistent with Hu et al.’s findings, indicating that nearly half of the Asian population falls under Roussouly type 3 [47]. Typically, these higher Roussouly types are known for balanced lordosis distribution, potentially impacting force dynamics at the L5/S1 segment [46]. Conversely, Qu et al. found a tendency for anterolisthesis or disc space narrowing in ASD among patients with Roussouly type 2 and high pelvic tilt [40]. Thus, further research is essential to establish a clear correlation between Roussouly types and the development of ASD at the L5-S1 level.

During the initial period of 1 year, representing the duration of both fusions, the survival curve shows no difference between patients exhibiting facet effusion and those without, as well as between patients with and without severe disc degeneration, followed by a decline with a consistent slope (Figure 3a,b). These results imply that the preoperative presence of facet effusion and severe disc degeneration at the L5-S1 level have both mid-term and long-term impacts on symptomatic ASD development in L5-S1 following OLIF at the L4-L5 level and above. The subsequent gradual decline with no plateau indicates the disease course of progressive symptomatic ASD after OLIF of the L4-L5 level and above, which is consistent with Gao’s study demonstrating a longer follow-up period as a risk factor for ASD [16].

In contrast to facet effusion and disc degeneration, patients with severe fatty degeneration of the paraspinal muscle exhibited an initial steep drop in the survival curve, which subsequently transitioned to a more gradual decline (Figure 3c). This implies that severe preoperative fatty degeneration of the paraspinal muscle could be the result of a hidden pre-existing L5 or S1 radiculopathy. Hidden L5 or S1 radiculopathy may contribute to the progression of fatty degeneration due to paraspinal muscle denervation, as evidenced by Hodges’ study showing an association between root injuries and the multifidus cross-sectional area [48].

This study has several limitations. First, the criterion for radicular pain necessitating ESI, which was established as an outcome measure, may be subjective. Consequently, this may have resulted in the exclusion of a considerable number of patients showing failure of conservative treatment. However, many studies have shown that radiculopathy and spinal stenosis at the adjacent segment are the main symptoms of symptomatic ASD after fusion [6,49,50,51]. According to a recent review, 72% of clinical ASD studies employed criteria such as radiculopathy or neurological signs [52]. Other studies in the review used new symptoms requiring further interventions like revision surgery as clinical ASD indicators. These additional treatments denote conservative management failure, which our study also aimed to identify, choosing the incidence of L5 or S1 radicular pain necessitating ESI as a key criterion. Second, the retrospective design of this study is inherently susceptible to selection bias. Third, an evaluation of clinical scores, including the visual analog scale, Oswestry disability index, and Japanese Orthopaedic Association Back Pain Evaluation Questionnaire, was not performed. However, the current study originally aimed to identify the risk factors associated with symptomatic ASD at the L5-S1 level in the presence of preoperative degenerative changes. Therefore, excluding these factors was not expected to alter the outcome of our analysis substantially. Fourth, an evaluation of fusion status and cage subsidence was not performed. Fusion status and cage subsidence can provide much more information about adjacent segment disease. However, none of the patients in our study required additional surgery for nonunion or cage subsidence; therefore, we believe that omitting this information did not impact our results.

## 5. Conclusions

In this study, patients who presented with preoperative facet effusion in the L5-S1 facet joint, severe degeneration of the L5-S1 intervertebral disc, or severe degeneration of the paraspinal muscles showed a higher risk of symptomatic ASD at the L5-S1 level after OLIF of L4-L5 and above than patients without. The preoperative presence of facet effusion and severe disc degeneration at the L5-S1 level has both mid-term and long-term impacts on symptomatic ASD development at the L5-S1 level following OLIF of the L4-L5 level and above. Facet effusion could serve as a better index of instability at the L5-S1 level, compared to the prevailing instability criteria. Therefore, surgeons may consider incorporating the L5-S1 level in fusion surgery to improve the durability of surgical outcomes in patients presenting with these findings.

## Figures and Tables

**Table 1:** Baseline characteristics of included patients.

Baseline Characteristics	All Patients		
	ASD Group (*n* = 34)	Non-ASD Group (*n* = 157)	*p*-Value
Age (mean ± SD, years)	69.3 ± 6.8	68.5 ± 8.6	0.595
Sex, Male (*n* (%))	13 (38.2%)	59 (37.6%)	0.943
BMI (mean ± SD, kg/m^2^)	25.0 ± 3.4	25.1 ± 3.0	0.861
Follow-up period (mean ± SD, month)	45.8 ± 18.4	42.1 ± 17.3	0.267
Time from OLIF to L5 ESI (mean ± SD, month)	19.9 ± 20.9		
Preoperative diagnosis (*n* (%))			0.463
Spinal stenosis without SPLT	11 (32.4)	38 (24.2)	
Degenerative SPLT	21 (61.8)	99 (63.1)	
Spondylolytic SPLT	1 (2.9)	10 (6.4)	
Adjacent segment disease	1 (2.9)	2 (1.3)	
Degenerative lumbar scoliosis	0 (0.0)	8 (5.1)	
The number of levels that underwent OLIF (*n* (%))			0.297
1 level	14 (41.2)	89 (56.7)	
2 levels	15 (44.1)	47 (29.9)	
3 levels	5 (14.7)	15 (9.6)	
4 levels	0 (0.0)	5 (3.2)	
5 levels	0 (0.0)	1 (0.6)	

SD, standard deviation; BMI, body mass index; OLIF, oblique lumbar interbody fusion; ESI; epidural steroid injection; SPLT, spondylolisthesis.

**Table 2:** Comparison of radiologic parameters between the ASD group and non-ASD group.

Radiologic Parameters	All Patients		
	ASD Group (*n* = 34)	Non-ASD Group (*n* = 157)	*p*-Value
Disc vacuum (*n* (%))	3 (8.8%)	4 (2.5%)	0.077
Calcified L5-S1 disc (*n* (%))	3 (9.1%)	30 (19.1%)	0.150
Deep-seated L5 (*n* (%))	10 (29.4%)	55 (35.0%)	0.531
PI (mean ± SD, degree)	54.3 ± 8.9	54.6 ± 8.6	0.850
Preoperative sacral slope (mean ± SD, degree)	33.2 ± 5.6	33.8 ± 7.8	0.586
Preoperative pelvic tilt (mean ± SD, degree)	21.1 ± 8.7	21.2 ± 7.5	0.936
Preoperative LDI (mean ± SD, percentage)	70.9 ± 26.9	76.0 ± 81.0	0.714
Postoperative LDI (mean ± SD, percentage)	64.0 ± 18.4	68.1 ± 36.8	0.530
Preoperative PI minus LL (mean ± SD, degree)	12.2 ± 13.8	12.4 ± 13.4	0.937
Postoperative PI minus LL (mean ± SD, degree)	10.2 ± 10.3	8.2 ± 9.7	0.292
Preoperative SVA (mean ± SD, degree)	33.8 ± 43.2	34.5 ± 47.9	0.941
Postoperative SVA (mean ± SD, degree)	20.6 ± 38.3	25.4 ± 33.5	0.468
Preoperative flexion–extension ROM in L5-S1 disc (mean ± SD, degree)	8.0 ± 4.3	6.7 ± 4.3	0.113
Instability in L5-S1 level (*n* (%))	10 (30.3%)	39 (24.8%)	0.514
Lee grade 1 foraminal stenosis (*n* (%))	7 (20.6%)	33 (21.0%)	0.955
Bartynski grade 1 lateral recess stenosis (*n* (%))	8 (23.5%)	24 (15.3%)	0.243
Pfirrmann grade at L5-S1 disc (*n* (%))			0.102
Grade I	1 (2.9%)	9 (5.7%)	
Grade II	7 (20.6%)	30 (19.1%)	
Grade III	11 (32.4%)	80 (51.0%)	
Grade IV	12 (35.3%)	31 (19.7%)	
Grade V	3 (8.8%)	7 (4.5%)	
Preoperative grade of facet arthrosis in L5-S1 facet joint (*n* (%))			0.120
Grade 0	3 (8.8%)	34 (21.7%)	
Grade 1	14 (41.2%)	56 (35.7%)	
Grade 2	13 (38.2%)	57 (36.3%)	
Grade 3	4 (11.8%)	10 (6.4%)	
Facet effusion in L5-S1 level (*n* (%))	13 (38.2%)	30 (19.1%)	0.015
Goutallier grade of paraspinal muscle in L5-S1 disc level (*n* (%))			0.014
Grade 0	0 (0.0%)	3 (1.9%)	
Grade 1	4 (11.8%)	31 (19.7%)	
Grade 2	21 (61.8%)	109 (69.4%)	
Grade 3	6 (17.6%)	7 (4.5%)	
Grade 4	3 (8.8%)	7 (4.5%)	

ASD, adjacent segment disease; PI, pelvic incidence; LDI, lordosis distribution index; LL, lumbar lordosis; SD, standard deviation; SVA, sagittal vertical axis; ROM, range of motion.

**Table 3:** Univariate and multivariate logistic regression analyses of symptomatic ASD at the L5-S1 level.

	Univariate Analysis		Multivariate Analysis	
	Odds Ratio (95% CI)	*p* Value	Odds Ratio (95% CI)	*p* Value
Multi-level OLIF ^a^		0.103		0.098
Disc vacuum		0.097		0.563
Calcified L5-S1 disc		0.162		0.316
Preoperative flexion–extension ROM in L5-S1 disc		0.115		0.082
Pfirrmann grade ≥ IV in L5-S1 disc	2.472 (1.146–5.335)	0.021	2.653 (1.156–6.091)	0.021
Facet effusion at the L5-S1 level	2.621 (1.180–5.820)	0.018	2.553 (1.046–6.231)	0.040
Goutallier grade ≥ 3 of paraspinal muscle at the L5-S1 disc level	3.677 (1.438–9.404)	0.007	4.473 (1.533–13.047)	0.006

^a^ Odds compared to single level. CI, confidence interval; ROM, range of motion.

## Data Availability

The data underlying this article cannot be shared publicly due to the privacy of the individuals that participated in this study. The data may be shared on reasonable request to the corresponding author.

## Supplementary Materials

The following supporting information can be downloaded at: https://www.mdpi.com/article/10.3390/jcm12237463/s1, Table S1: Full version of univariate and multivariate logistic regression analyses about symptomatic ASD in L5-S1 level after OLIF of L4-L5 level and above; Table S2: Likelihood ratio test in mediation analysis to identify interaction effects among facet effusion, severe fatty degeneration, and severe disc degeneration.
