# Supplementary material for: The Fate of Pre-Existing L5-S1 Degeneration following Oblique Lumbar Interbody Fusion of L4-L5 and Above
Source: J Clin Med. 2023 Dec 1;12(23):7463. doi: 10.3390/jcm12237463 (PMC10707249; doi:10.3390/jcm12237463)
Supplement: Supplementary file 1 [file jcm-12-07463-s001.zip › jcm-2719473-supplementary.pdf]

Table S1. Full version of univariate and multivariate logistic regression analyses about symptomatic ASD in L5-S1 level after OLIF of L4-L5 level and above.

	Univariate analysis		Multivariate analysis	
	Odds ratio (95% CI)	*P* value	Odds ratio (95% CI)	*P* value
Age (years)		0.593		
Sex (male)		0.943		
BMI		0.860		
Multi-level OLIF [a]		0.103		0.098
Revision		0.504		
Disc vacuum		0.097		0.563
Calcified L5-S1 disc		0.162		0.316
Deep seated L5		0.531		
Preoperative PI minus LL		0.937		
Preoperative PI minus LL ≥ 15		0.357		
Postoperative PI minus LL		0.290		
Postoperative PI minus LL ≥ 15		0.688		
Preoperative SVA		0.940		
Postoperative SVA		0.466		
Preoperative flexion-extension ROM in L5-S1 disc		0.115		0.082
Instability in L5-S1 disc		0.515		
Decreased disc height		0.786		
Anterior disc height in MRI		0.749		
Posterior disc height in MRI		0.860		
Grade 1 foraminal stenosis in L5-S1 level		0.955		
Grade 1 lateral recess stenosis in L5-S1 level		0.247		
Pfirrmann grade ≥ IV at L5-S1 disc	2.472 (1.146-5.335)	0.021	**2.653 (1.156-6.091)**	**0.021**
Facet arthropathy in L5-S1		0.323		
Grade 1[b]		0.121		
Grade 2[b]		0.160		
Grade 3[b]		0.073		
Grade 3 facet arthropathy in L5-S1		0.281		
Facet effusion in L5-S1 level	2.621 (1.180-5.820)	0.018	**2.553 (1.046-6.231)**	**0.040**
Goutallier grade ≥ 3 of paraspinal muscle at L5-S1 disc level	3.677 (1.438-9.404)	0.007	**4.473 (1.533-13.047)**	**0.006**
[a] Odds compared to single level, [b] Odds compared to grade 0				

BMI, body mass index; OLIF, oblique lumbar interbody fusion; LL, lumbar lordosis; PI, pelvic incidence; SVA, sagittal vertical axis; ROM, range of motion

Table S2. Likelihood ratio test in mediation analysis to identify interaction effects among facet effusion, severe fatty degeneration, and severe disc degeneration.

Combination of variables		Coefficient of the predictor variable	Standard error	*P*	95% CI	
					LL	UL
Facet effusion	Severe disc degeneration	-0.6293	0.8334	0.4502	-2.2627	1.0042
Facet effusion	Severe fatty degeneration	0.1844	1.3876	0.8943	-2.5352	2.9040
Severe fatty degeneration	Severe disc degeneration	-0.4406	1.0594	0.6775	-2.5170	1.6358

P <0.05 signifies a significant interaction between the two variables.
